# Lipid remodelling in the reef-building honeycomb worm, *Sabellaria alveolata*, reflects acclimation and local adaptation to temperature

**DOI:** 10.1038/srep35669

**Published:** 2016-10-20

**Authors:** Anna P. Muir, Flavia L. D. Nunes, Stanislas F. Dubois, Fabrice Pernet

**Affiliations:** 1 Laboratoire des Sciences de l’Environnement Marin, LEMAR UMR 6539 CNRS/UBO/IRD/Ifremer, Université de Brest (UBO), Université Européenne de Bretagne (UEB), Institut Universitaire Européen de la Mer (IUEM), 29280 Plouzané, France; 2 Department of Biological Sciences, University of Chester, Parkgate Road, Chester, CH1 4BJ, UK; 3 Ifremer Centre Bretagne, DYNECO, Laboratoire d’Écologie Benthique Côtière (LEBCO) 29280 Plouzané, France; 4 Ifremer Centre Bretagne, PFOM, Laboratoire de Physiologie des Invertébrés (LPI), UMR 6539 (CNRS, UBO, IRD, IFREMER), Laboratoire des Sciences de l’Environnement Marin (LEMAR), 29280 Plouzané, France

## Abstract

Acclimation and adaptation, which are key to species survival in a changing climate, can be observed in terms of membrane lipid composition. Remodelling membrane lipids, via homeoviscous adaptation (HVA), counteracts membrane dysfunction due to temperature in poikilotherms. In order to assess the potential for acclimation and adaptation in the honeycomb worm, *Sabellaria alveolata*, a reef-building polychaete that supports high biodiversity, we carried out common-garden experiments using individuals from along its latitudinal range. Individuals were exposed to a stepwise temperature increase from 15 °C to 25 °C and membrane lipid composition assessed. Our results suggest that *S. alveolata* was able to acclimate to higher temperatures, as observed by a decrease in unsaturation index and 20:5n-3. However, over the long-term at 25 °C, lipid composition patterns are not consistent with HVA expectations and suggest a stress response. Furthermore, unsaturation index of individuals from the two coldest sites were higher than those from the two warmest sites, with individuals from the thermally intermediate site being in-between, likely reflecting local adaptation to temperature. Therefore, lipid remodelling appears limited at the highest temperatures in *S. alveolata*, suggesting that individuals inhabiting warm environments may be close to their upper thermal tolerance limits and at risk in a changing climate.

Ongoing climate change is predicted to have important consequences for ectotherms, due to the direct influence of the thermal environment on metabolic rates, growth, reproduction and locomotion^1^,^2^. This is applicable to ectotherms in marine environments, where body temperatures closely track surrounding water temperatures and where thermal profiles influence species distributions^3^. Furthermore, marine organisms inhabiting the intertidal zone are subject to extreme temperature fluctuations on a daily basis due to their exposure to both water and air temperatures at high- and low-tide, respectively^4^. However, the mechanisms that allow survival in such challenging environments are often unclear and could be due to behavioural responses, genetic adaptation and/or phenotypic plasticity^5^,^6^. Therefore, organisms inhabiting the intertidal zone provide an excellent opportunity to understand the mechanisms underlying tolerance to a broad range of temperatures, which is key when evaluating the potential effects of climate change^4^. For instance, warm-adapted porcelain crabs (genus *Petrolisthes*), intertidal limpets, and snails of the genus *Chlorostoma* are actually more threatened by ocean warming than their cold water congeners as they are already living close to their upper thermal tolerance limits^7^,^8^,^9^. Furthermore, the species of porcelain crabs that have adapted to high temperatures have done so at the expense of acclimation capacity^7^. Therefore, assessing the interplay between adaptation and acclimation in responding to novel temperatures in littoral zone species can increase our understanding of the impacts of a changing climate.

Adaptation, an evolutionary response across generations, and acclimatisation, here defined as any response in which a genotype exhibits different phenotypes in response to the environment experienced, are both important facets of survival in a changing climate^6^. However, the rapidity of anthropogenically-induced climate change has highlighted acclimatisation as particularly important in short-term species survival, whilst adaptation will likely predict survival in the long-term^5^. Comparing the response of populations of the same species that experience different thermal regimes, allows the role of adaptation and acclimatisation in species survival to be untwined (for reviews see refs 5 and 10).

Cellular membrane organisation is key to maintaining physiological function because membranes act as physical barriers to diffusion, control the movement of transmembrane solutes, regulate energy usage in terms of transmembrane ion gradients, and structure and regulate signal pathways^11^. However, temperature can alter membrane organisation by modifying fluidity, potentially leading to membrane dysfunction^11^. Ectothermic animals usually counteract the effect of temperature on membrane fluidity by remodelling membrane lipids, a process known as homeoviscous adaptation (HVA)^12^,^13^. HVA involves changes in phospholipid headgroups, fatty acid composition and cholesterol content, resulting in the biosynthesis of membranes with equivalent viscosities regardless of the temperature experienced^13^. The unsaturation index (UI) is a measure of the number of double bonds within a membrane lipid sample and can be used to assess the relationship between HVA and temperature^14^. Acclimation to lower temperatures is typically followed by an increase in acyl chain unsaturation in cellular membranes in a wide range of organisms^11^. For example, long chain polyunsaturated fatty acids increase with decreasing acclimation temperature in gill phospholipids of the sea scallop, *Placopecten magellanicus*^15^. Acclimation to high temperatures has a correspondingly similar effect in the Eastern oyster, *Crassostrea virginica*, and blue mussel, *Mytilus edulis*: UI of membrane phospholipids decreases as temperature increases^16^. Furthermore, *M. edulis,* which naturally inhabits a colder environment than *C. virginica,* shows higher UI when brought into a common environment, indicating adaptation^16^. Differentiation between thermal adaptation and acclimatisation responses can therefore be observed using membrane lipid remodelling.

The honeycomb worm, *Sabellaria alveolata*, is a reef-building polychaete that inhabits the intertidal zone and supports a wide range of biodiversity in its role as an ecosystem engineer; they are consequently of high conservation concern^17^. Biogenic reefs, including *Sabellaria* species, are listed under the EC Habitats Directive (92/43/EEC) Annex 1. *S. alveolata* occurs along a latitudinal gradient from the Irish Sea in Scotland to North Africa, thus experiencing a range of environmental conditions within its distribution^18^,^19^. Temperature ranges from a mean summer sea surface temperature of 13.7 °C at the northernmost point of the range in the Irish Sea to 23.0 °C at the most southerly location, in North Africa^20^. However, temperature does not vary linearly with latitude, instead, upwelling along the Iberian coast creates a cold zone^21^. This non-linearity in the environmental gradient offers an excellent opportunity to relate adaptation to temperature, rather than to other environmental factors correlated with latitude. Reefs are exposed to air and water temperatures during the course of tidal cycles, meaning that reefs are also subject to high temperature fluctuations on a daily basis, making them an ideal candidate for studying responses to changes in temperature^4^. Specifically, *S. alveolata* can experience daily temperature fluctuations of up to 20 °C^22^ and must thus be able to respond to a wide range of temperatures that are experienced over the tidal cycle. However, the role of acclimatisation and adaptation in surviving in differential thermal environments has not yet been explored in this foundation species.

In order to assess the potential for *S. alveolata* to adapt or acclimatise to increasing ocean temperatures, we carried out common-garden experiments using individuals sampled along the latitudinal range of this species. Reef cores of *S. alveolata* were exposed to a stepwise temperature increase from 15 °C to 25 °C, a temperature range normally encountered by this species along its distribution area. We predicted that: 1) *S. alveolata* would counteract thermal effects by changing acyl chain unsaturation of phospholipids as stipulated by the HVA, thereby acclimating to different temperatures; and 2) individuals from warmer sites would show a lower proportion of unsaturated fatty acids in phospholipids, suggesting local adaptation.

## Results

### Hierarchical clustering

**Figure 1:**
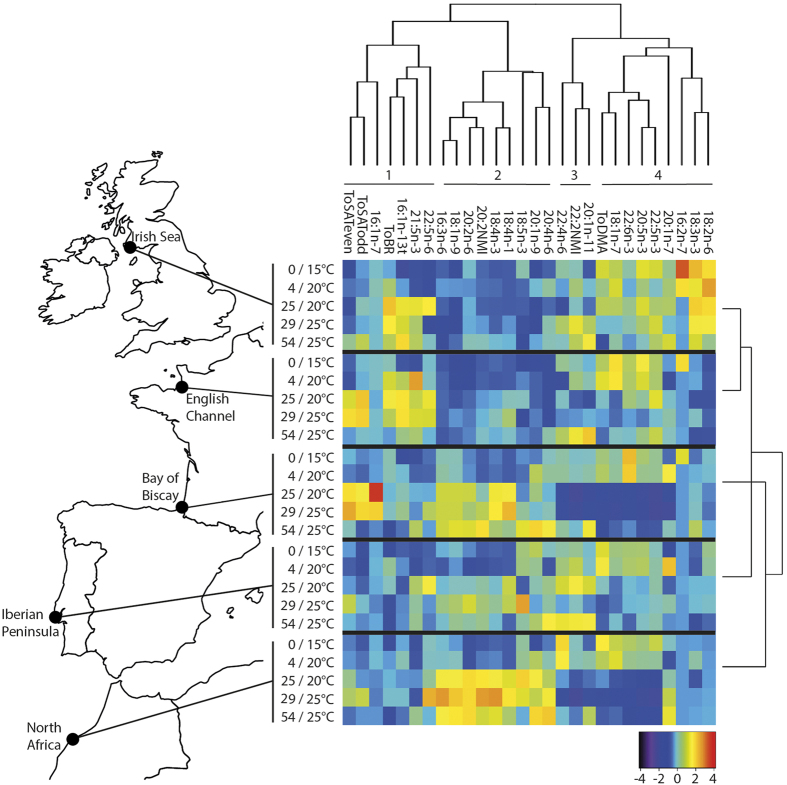
Heat map showing lipids contributing >1 mol% of total fatty acids averaged by sampling time. Fatty acids were reordered according to the hierarchical clustering result given by the dendrogram at the top and clusters identified using gap statistics. The dendrogram on the right shows how the lipid profiles cluster by site. Sample sites are shown on the left (map created using the package rworldmap^60^ in R version 3.1.2^58^). Sampling points are shown in days/temperature. The abbreviation ToSATeven was used for total even-chain saturated fatty acids, ToSATodd for total odd-chain saturated fatty acids, ToBR for total branched fatty acids and ToDMA for total dimethylacetals.

Four fatty acid clusters were identified among all sampled individuals in the five sites (Fig. 1). Cluster 1 groups seven fatty acids and fatty acid groups, some of them being of bacterial origin such as odd and branched fatty acids, 16:1n-7 and trans-16:1n-13^23^. Cluster 2 includes 20:4n-6, 20:2 non-methylene interrupted (20:2 NMI) and seven other miscellaneous minor fatty acids. 20:4n-6 is a precursor of eicosanoids^24^, a group of hormones associated with stressful situations, such as gametogenesis and spawning in bivalves or stimulation of immune function in other invertebrates^25^,^26^. NMI fatty acids originate from unusual biosynthetic pathways in molluscs and their biological function is presently not well-known^27^. Cluster 3 is composed of 22:4n-6 plus 20:1n-11 and 22:2 NMI, two fatty acids that are often associated with plasmalogens in marine bivalves^27^,^28^. Finally, Cluster 4 includes nine fatty acids, including long chain n-3 polyunsaturated fatty acids (PUFAs) such as 20:5n-3, 22:5n-3 and 22:6n-3. This group also includes 18:2n-6 and 18:3n-3, two markers of terrestrial inputs and vascular plants^23^,^29^. Finally, this cluster includes dimethylacetals (DMA), which reflect plasmalogen concentration^28^.

The lipid profiles of individuals from the Irish Sea and the English Channel clustered together, and were then most similar to that of individuals from the Iberian Peninsula, and the lipid profiles of individuals from the Bay of Biscay and North Africa clustered together (Fig. 1). This clustering pattern is in line with the temperatures of the five sites: the Irish Sea and the English Channel have the coldest mean summer sea surface temperatures, followed by the Iberian Peninsula, then the Bay of Biscay and North Africa (Table 1).

### Effect of temperature and site

**Figure 2:**
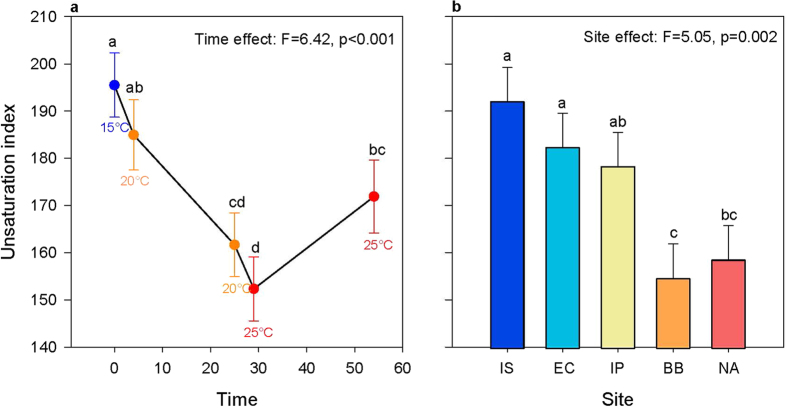
Plots showing the significant effect of time (days; **a**) and of site (**b**) on unsaturation index. Unsaturation index was averaged over all sites for each time point in (**a**), while the average over all timepoints are shown for each population in (**b**). Letters are used to show pairwise significant differences between timepoints or sites such that if no letters are shared between two categories then there is a significant difference between them. The abbreviation IS is used for Irish Sea, EC for English Channel, IP for Iberian Peninsula, BB for Bay of Biscay and NA for North Africa. Sites are ordered from left to right on the axis from coldest to warmest.

The UI of polar lipids corresponds to the number of double bonds per mole of a fatty acid, and is an indicator of membrane fluidity^12^,^13^,^14^. In our study, site and time but not their interaction were significant in their effect on UI (Fig. 2, also see Supplementary Fig. S1). UI of *S. alveolata* decreased during the first 29 days while temperature increased from 15 °C to 25 °C (Fig. 2a). Then, UI increased between 29 days and 54 days during long-term exposure to 25 °C. Also, the coldest sites (Irish Sea and English Channel) showed higher average UI across all sampling points combined than the warmest sites (Bay of Biscay and North Africa), with average UI in the Iberian Peninsula being intermediate (Fig. 2b).

**Figure 3:**
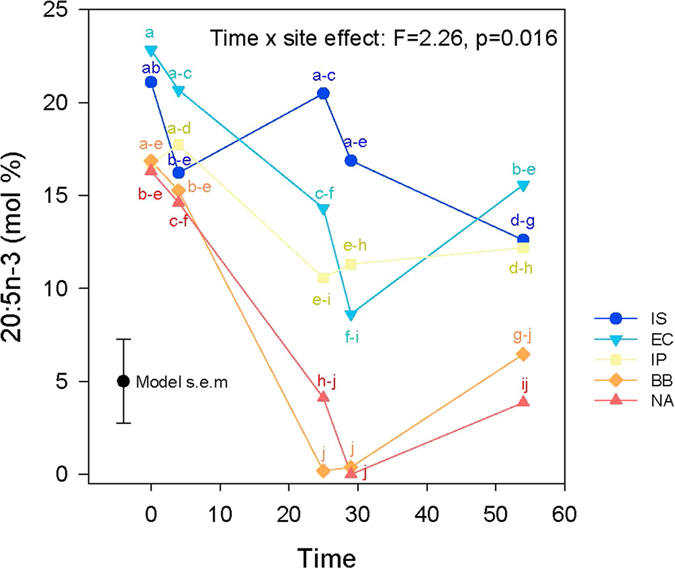
Plot showing the significant effect of the interaction between time and site on 20:5n-3. Letters are used to show pairwise significant differences such that if no letters are shared between two points then there is a significant difference between them. Standard error of the model is shown (Model s.e.m). The abbreviation IS is used for Irish Sea, EC for English Channel, IP for Iberian Peninsula, BB for Bay of Biscay and NA for North Africa.

The multiple regression analysis revealed that 20:5n-3 explained 85% of the variation in UI among sites and time points (see Supplementary Table S1). This fatty acid showed a significant effect of the interaction between site and time (Fig. 3) with individuals from the Bay of Biscay and North Africa showing the most pronounced pattern of change with time, such that 20:5n-3 decreased as temperature increased from 15 °C to 20 °C but then increased again after exposure to 25 °C. This was also seen to a lesser extent in individuals from the English Channel and Iberian Peninsula (Fig. 3). Individuals from the Irish Sea showed no clear pattern of change, although, as with the other sites, 20:5n-3 was lower following exposure to 25 °C than at 15 °C (Fig. 3).

**Figure 4:**
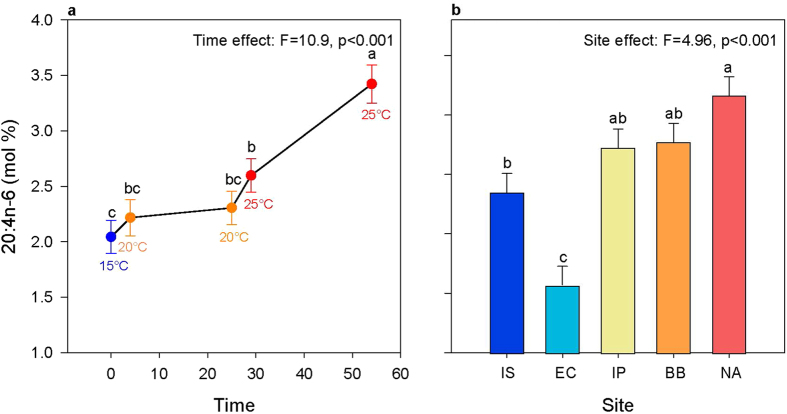
Plots showing the significant effect of time (days; **a**) and of site (**b**) on 20:4n-6. Unsaturation index was averaged over all sites for each time point in (**a**), while the average over all timepoints are shown for each population in (**b**). Letters are used to show pairwise significant differences between timepoints or sites such that if no letters are shared between two categories then there is a significant difference between them. The abbreviation IS is used for Irish Sea, EC for English Channel, IP for Iberian Peninsula, BB for Bay of Biscay and NA for North Africa. Sites are ordered from left to right on the axis from coldest to warmest.

The fatty acid 20:4n-6, which ranked second in explaining an additional 6% of the change in UI (see Supplementary Table S1) showed a significant effect of site and time but not their interaction. 20:4n-6 increased slowly with temperature and time but showed the most pronounced increase after exposure to 25 °C for 25 days (Fig. 4a). This fatty acid was significantly lower in individuals from cold sites (Irish Sea and English Channel) compared to those from the warmest site (North Africa). Individuals from the Bay of Biscay and the Iberian Peninsula were intermediate, such that levels in individuals from the Bay of Biscay and the Iberian Peninsula were not significantly different to levels in the individuals from the Irish Sea or North Africa (Fig. 4b).

**Figure 5:**
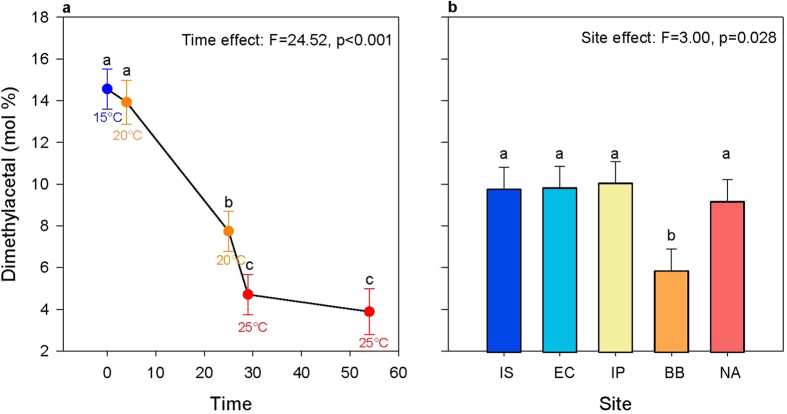
Plots showing the significant effect of time (days; **a**) and of site (**b**) on dimethylacetal. Unsaturation index was averaged over all sites for each time point in **(a**), while the average over all timepoints are shown for each population in (**b**). Letters are used to show pairwise significant differences between timepoints or sites such that if no letters are shared between two categories then there is a significant difference between them. The abbreviation IS is used for Irish Sea, EC for English Channel, IP for Iberian Peninsula, BB for Bay of Biscay and NA for North Africa. Sites are ordered from left to right on the axis from coldest to warmest.

Total DMA showed a significant effect of site and time but not their interaction and decreased with time and temperature (Fig. 5a). Total DMA was also lower in individuals from the Bay of Biscay compared to other sites (Fig. 5b). The fatty acids 22:2NMI and 20:1n-11 showed a significant effect of the interaction between site and time (see Supplementary Figs S2 and S3). Individuals from the Bay of Biscay, North Africa and the English Channel showed the clearest pattern of change in these two fatty acids with time, specifically, levels gradually decreased over the first 29 days before increasing again following 25 days exposure to 25 °C.

The fatty acid 20:2NMI also showed a significant effect of the interaction between site and time but it behaved differently from 22:2NMI (see Supplementary Fig. S4). This fatty acid increased markedly in individuals from North Africa, followed by those from the Bay of Biscay and the Iberian Peninsula, and it remained fairly constant in polychaetes from the English Channel and the Irish Sea.

Branched fatty acids showed a positive relationship with time between 15 °C and 20 °C, followed by a negative relationship following long-term exposure to 25 °C (see Supplementary Fig. S5). Branched fatty acids were higher in individuals from the colder sites (Irish Sea and English Channel) than the warmer sites (Iberian Peninsula, Bay of Biscay and North Africa) (see Supplementary Fig. S5). All raw fatty acid data is available in the Supplementary Dataset.

### Occupancy

**Figure 6:**
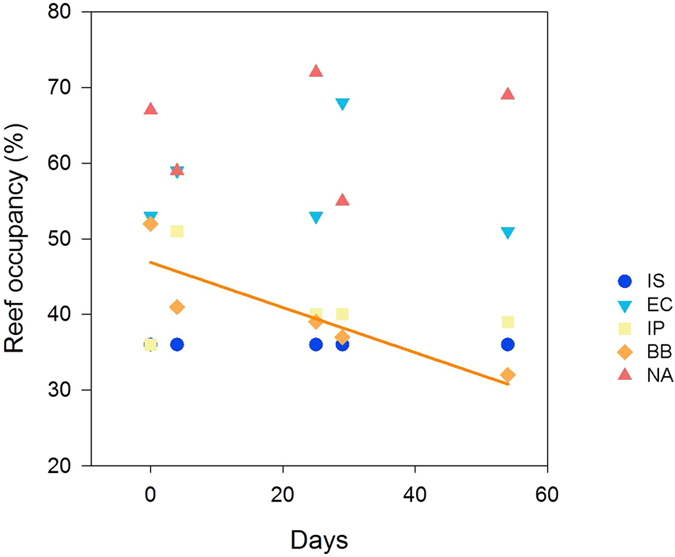
Percentage of tubes that were observed as occupied for each site at each timepoint. The linear regression line for the Bay of Biscay is shown as occupancy had a significant relationship with time in individuals from this site (p = 0.01). The abbreviation IS is used for Irish Sea, EC for English Channel, IP for Iberian Peninsula, BB for Bay of Biscay and NA for North Africa.

Reef occupancy did not significantly regress with time for four of the five sites at the Irish Sea (p = 1.0), English Channel (p = 0.73), Iberian Peninsula (p = 0.50) and North Africa (p = 0.50). In contrast, occupancy had a significant negative relationship with time in individuals from the Bay of Biscay (p = 0.01; Fig. 6).

## Discussion

Our first hypothesis, that *S. alveolata* would counteract thermal effects by changing acyl chain unsaturation of phospholipids as stipulated by the HVA, thereby acclimating to different temperatures, was supported by the data. The acyl chain unsaturation of phospholipids of *S. alveolata* from all sites decreased markedly during the first 29 days while temperature increased from 15 °C to 25 °C. Although membrane fluidity was not measured in our study, this temporal decrease in UI may have counteracted the disordering effect of rising temperatures in a way consistent with HVA^11^,^13^. Lipid restructuring is unquestionably one of the most common responses to temperature variation in ectotherms as it has been documented in many major taxonomic groups such as fishes^14^,^30^, marine invertebrates^15^,^16^ and insects^31^,^32^.

Intriguingly, low UI was not maintained over the long term at 25 °C. This response may reflect three possible explanations. First, the polychaetes may have used other unmeasured compensatory acclimation mechanisms for maintaining membrane fluidity. Indeed, HVA relies not only on acyl chain unsaturation, but also on changes of phospholipid headgroups^33^ and cholesterol content^34^. The rise in UI could therefore reflect a switch from acyl chain unsaturation to another mechanism that maintains fluidity. A second possibility is that the polychaetes were not able to maintain lipid saturation, leading to the observed increase in UI, and thus lack of continued acclimation. Lipid remodelling may be costly, and a slowdown in acyl chain saturation could lead to increases in UI because their dietary supply of fatty acids (phytoplankton) is highly unsaturated^16^. Finally, UI is also a summary of multiple fatty acids that have different dynamics and functions. Changes in UI were predominantly due to 20:5n-3, but 20:4n-6 was also an important contributor. While 20:5n-3 remained stable at 25 °C (Fig. 3a), 20:4n-6 increased markedly (Fig. 4a). The fatty acid 20:5n-3 is known for regulating membrane fluidity in scallops^15^, and both 20:5n-3 and 20:4n-6 are precursors of eicosanoids, a group of biologically active metabolites involved in stress response^24^. Therefore, membrane fluidity may have been maintained (by 20:5n-3) while the increase in UI was primarily related to increases in 20:4n-6, indicating a stress response at 25 °C.

Global fatty acid profiling grouped 20:5n-3, thought to be important in membrane dynamics and temperature acclimation, with DMA. The presence of DMA indicates the presence of plasmalogens in polar lipids. In our study, the level of DMA decreased markedly with temperature in *S. alveolata*, as previously reported in mitochondria of the clam *Spisula solidissima* when temperature increased from overwintering to summer^35^. However, these results disagree with studies conducted on nervous tissues and mitochondria of fishes where plasmalogens are positively correlated with temperature^36^,^37^. Also, the level of ethanolamine plasmalogens is 20% higher in whole tissues of amphipods *Gammarus* spp. acclimated at 15 °C compared to those at 8 °C^38^. These studies are consistent with the idea that plasmalogens decrease membrane fluidity^39^ and therefore increase in warm-adapted organisms. However, plasmalogens form non-lamellar structures at lower temperatures than their diacyl homologs^40^. Therefore, their reduced levels may prevent phase transition in membranes of *S. alveolata* and thus maintain a bi-lamellar structure. To our knowledge, *S. alveolata* is one of the only examples of a marine organism where DMA decreases markedly with temperature resulting in unusually low levels of plasmalogens^37^.

The level of 20:4n-6 slightly increased with temperature between 15 °C and 25 °C followed by marked increase during long-term acclimation at 25 °C, in agreement with patterns observed in bivalves^16^ and crustaceans^41^. Particularly, the cold-adapted mussel *M. edulis* shows a higher rate of increase in 20:4n-6 in their polar lipids than the warm-adapted oyster *C. virginica* when temperature increased from overwintering to spring^16^. The fatty acid 20:4n-6 is a precursor of hormones involved in stress response. Therefore, increasing levels of 20:4n-6 with temperature in *S. alveolata* likely reflect a stress response as reported in other cold-adapted species.

Overall, our results suggest that *S. alveolata* was able to acclimate to a high temperature over short-term as observed by the decrease in UI and 20:5n-3 (Fig. 2). However, over the long-term at 25 °C, patterns of UI and 20:5n-3 are not consistent with HVA expectations and patterns of 20:4n-6 and DMA suggest a stress response. The potential for short-term acclimation to high temperature can be understood based on the ecology of the species. Species inhabiting the mid-level littoral zone are exposed to daily temperature fluctuations of up to 20 °C^22^ and various coping mechanisms have evolved to withstand such variable environments. Some intertidal species can moderate their temperature through behavioural responses^42^ but, as sessile adults, *S. alveolata* have limited changes in behaviour to avoid high temperatures, such as retracting into the tube or retaining water inside the tube. As a result, HVA is possibly an important mechanism for acclimating to high temperatures in *S. alveolata*. However, long term exposure to temperatures of 25 °C or greater may be outwith the usual experience of *S. alveolata,* as such temperatures are normally only experienced for the length of a low tide. Thermal tolerance experiments would help to elucidate how temperature is likely to affect the physiology of this organism and to improve predictions about how increasing global temperatures may affect *S. alveolata* across its range. Furthermore, future experiments should use temperature profiles that reflect the daily fluctuations experienced by *S. alveolata* to further elucidate the interplay between responses to short- and long-term exposure to high temperatures.

Our second hypothesis, that individuals from warmer sites would show a lower proportion of unsaturated fatty acids in phospholipids, suggesting local adaptation, was also supported by the data. The UI of individuals from the two coldest sites (Irish Sea and English Channel) were higher than those from the warmest sites (Bay of Biscay and North Africa), with individuals from the Iberian Peninsula being intermediate (Fig. 2). These differences in UI likely reflect local adaptation to temperature, with higher UI values indicating adaptation to lower temperature. For instance, UI of the cold-adapted mussel *M. edulis* is higher than that of the warm adapted oyster *C. virginica*^16^. Also, there is a positive correlation between cold tolerance of *Drosophila* species and unsaturation of phospholipid fatty acids^32^. To our knowledge, our study is the first to report differences in UI that are consistent with thermal adaptation in a marine invertebrate along its latitudinal cline.

Furthermore, 20:5n-3, a fatty acid often involved in regulation of membrane fluidity^15^,^43^ and positively correlated with UI in our study, showed very little change in individuals from the northernmost site (Irish Sea) compared to individuals from other sites. Thus, the pattern of biochemical compensation in response to temperature in this species shows intraspecific variation consistent with adaptation. Differences in regulation of membrane fluidity were also observed between populations of the oyster *C. gigas* where changes in the UI in response to temperature were mainly due to 20:5n-3 levels in fast-growing hatchery animals, whereas changes in UI were driven by both 20:5n-3 and 22:6n-3 in slow-growing animals^44^.

Interestingly, 20:4n-6, a fatty acid related to stress response, was detected at lower levels in individuals from the English Channel and higher levels in those from North Africa. This may be related to the fact that the site at Mont St Michel in the English Channel has optimal conditions for the growth of *S. alveolata.* This site is central within the geographic distribution of *S. alveolata*, and is the location of the largest living biogenic reef in Europe^45^. In contrast, North Africa is a peripheral site situated near the southern distribution limit of the species, likely close to its thermal limit. Therefore, 20:4n-6 may potentially be a health index for *S. alveolata* reefs, such that low levels of 20:4n-6 may be indicative of good health. However, further studies are required to examine the relationship between 20:4n-6 and stress in *S. alveolata*, and how this fatty acid may be related to healthy or degraded sites in the field. Development of health indexes is useful for management of *S. alveolata*, providing a quantitative metric that can discern impacted areas (potentially averting irreversible damage) or for selecting areas of good health as candidates for marine protected areas and thus is worthy of further investigation.

The fatty acid 20:2NMI was fairly constant in polychaetes from the coldest sites (Irish Sea and English Channel) whereas it increased markedly with temperature in individuals from the warmest sites (North Africa and Bay of Biscay) and gradually in individuals from the intermediate site (Iberian Peninsula), likely reflecting local adaptation to temperature. In contrast, DMA was lowest in individuals from the Bay of Biscay, the only location where reef occupancy declined significantly during the experiment. Low DMA values may therefore indicate poor state of the reefs due to stressful thermal conditions. Indeed, plasmalogens (and therefore DMA) are antioxidant molecules that protect cell membranes from lipoxidation^46^. For example, the protective properties of plasmalogens have been inferred for clams, where plasmalogen levels decrease with ageing^35^, rendering older individuals more vulnerable to lipoxidation. Therefore, the contribution of plasmalogens to the polar lipids of *S. alveolata* may influence resistance to lipoxidation of biological membranes and its survival in warm environments.

Finally, branched fatty acids, which can also affect the physical properties of membranes^47^, were also higher in individuals from the colder than the warmer sites, indicating local adaption. However, temperature as the only selective force cannot be conclusively implied as individuals from the Iberian Peninsula, Bay of Biscay and North Africa did not have significantly different levels, meaning that we cannot rule out the selective influence of other factors related to latitude. Local adaptation in terms of these fatty acids is likely due to selective pressures other than or in addition to temperature.

In conclusion, acclimatisation and adaptation will predict short- and long-term species survival in a changing climate. Our results suggest that *S. alveolata* can acclimatise to the effects of increased temperature through HVA to maintain membrane fluidity and function and this ability is enhanced in individuals that have adapted to warm temperature environments. However, lipid remodelling appears limited at the highest temperatures in individuals from across the current range of *S. alveolata*, suggesting that individuals inhabiting warm environments may be close to their upper thermal tolerance limits. Therefore, the effects of ocean warming on reef health and survival, and how this will affect range shifts and/or local extinctions, deserve further investigations. This is an important finding given that *S. alveolata* are ecosystem engineers and their loss from an area would likely cause knock-on reductions in biodiversity.

## Methods

### Temperature challenge experiment

To investigate thermal acclimation and adaptation of *S. alveolata*, reef cores were collected from five locations spanning the latitudinal range of *S. alveolata* between March and April 2014 (Table 1). At each site, cores were collected from the middle of the cross-shore area colonised by *S. alveolata*. Cores were transported in cool boxes at 15 °C back to the laboratory at IFREMER, Brest, France, within a week of collection, where they were maintained within a recirculating seawater system, equipped with mechanical (50 μm) and ultraviolet (AquaCristal UV-C 9 W; JBL, Neuhofen, Germany) filtration. Worms were fed mixed live algal culture of *Isochrysis galbana* and *Skeletonema costatum*^48^ at a concentration of 644 ± 85 × 10^6^ μm^3^ mL^−1^ and 747 ± 152 × 10^6^ μm^3^ mL^−1^, respectively. Worms were acclimated to 15 ± 0.4 °C for four weeks prior to the commencement of the experiment. Water temperature was maintained using aquarium heaters and a flow rate of 120 L h^−1^ per 60 L site tank. Aeration and wave action was maintained using a Turbelle nanostream 6055 and a Wavecontroller 7092 (Tunze, Penzberg, Germany) per tank. Water temperature was monitored once a day to an accuracy of 0.1 °C and the light regime was set to 12 hours dark: 12 hours light.

At the start of the temperature challenge experiment, three individuals were extracted from the reef core from each site, rinsed and flash frozen in liquid nitrogen. Only individuals that had reached sexual maturity were sampled and maturation state was identified by the presence of gametes, as white (male) or purple (female) colouration of the worms is evident when they contain gametes^49^. Samples were stored at −80 °C. Following sampling, the water temperature within the tank system was gradually increased over a period of four days to 20 °C. Following one hour at 20 °C, three individuals were sampled as before to test for short-term acclimation responses to increased temperature. Water temperature was maintained at 20 ± 0.5 °C for 21 days and three individuals per site were sampled at the end of this period to test for long-term acclimation responses. Finally, temperature was gradually increased to 25 °C over the course of four days and three individuals per site were sampled after the temperature had reached 25 °C for one hour. Three individuals were again sampled following a period of 25 days at 25 ± 0.2 °C. The temperatures used in this experiment were chosen to be ecologically relevant as they reflect the mean summer sea surface temperatures that *S. alveolata* can be expected to experience within its latitudinal range (from 13.7 °C at Auchenmalg Bay, Scotland, to 23.0 °C at El Jadida, Morocco; Table 1). Therefore, at least one temperature treatment represents a thermal stress for individuals from each site.

At each time point, the reef cores were photographed from above three times, 25 tubes were randomly chosen, the number of tubes occupied by living worms were counted, and the total was averaged over the three images and converted to a mean percentage. Tubes were considered occupied by the presence of the white colour of the prostomium at the tube entrance^50^. Due to occupation being estimated from the number of observably occupied tubes and the fact that not all worms can be observed within their tubes at all times, it was possible for occupation % to increase slightly as well as decrease between times for each site. Decrease of the % occupancy is a proxy for mortality.

### Lipid profiling

Lipids were extracted from three *S. alveolata* whole individuals following Folch *et al.*^51^. The lipids were placed at the top of a silica gel microcolumn (30 × 5 mm internal diameter; Kieselgel; 70–230 mesh [Merck, Lyon, France]; previously heated to 450 °C and deactivated with 5% water). Neutral lipids were eluted with 10 mL of chloroform/methanol (98:2, v/v), and polar lipids were eluted with 15 mL of methanol^52^. Tricosanoic acid (2.3 μg) was added as internal standard. Polar lipids were transesterified at 100 °C for 10 min with 1 mL of boron trifluoride (12% Me–OH)^53^. This transesterification produces fatty acid methyl esters (FAME) from the fatty acid esterified at the sn-1 and sn-2 position of diacylphospholipids, and the sn-2 position of plasmalogen PL. It also produces dimethyl acetals (DMA) from the alkenyl chains at the sn-1 position of plasmalogens^54^. FAME and DMA were analysed in a HP6890 GC system (Hewlett-Packard) equipped with a DB-Wax capillary column (30 m × 0.25 mm; 0.25 μm film thickness; Agilent technologies). Peaks were analysed by comparison of their retention time with those of a standard 37 component fatty acid methyl ether (FAME) mix and other standard mixes from marine bivalves. Fatty acid contents were expressed as the mole percentage of the total fatty acid content. Total DMA was used as an indicator of the plasmalogen level.

### Statistical analyses

Levels of individual fatty acids were associated using a hierarchical clustering method based on the complete linkage with Euclidean distance measure and Ward’s linkage methods^55^. The optimal number of clusters was calculated with the gap statistic^56^ as implemented in the cluster package^57^ in R version 3.1.2^58^. Sites were also associated in terms of their fatty acid profiles. A heat map was generated to represent the data, where the relative abundances of the fatty acids were represented by squares with colour gradients. Only fatty acids >1 mole % in at least one treatment were included in this analysis. The multivariate analysis and heat map were performed using the gplots package^59^ in R^58^.

UI is a measure of the number of double bonds within a sample and it was calculated as the sum of the mole percentage of each unsaturated fatty acid multiplied by the number of double bonds within that fatty acid^14^. In order to rank the fatty acids in explaining the variation in UI, a stepwise regression model was calculated using UI as the dependent variable and each fatty acid as explanatory variable. The fatty acids found to explain the majority of the observed variation in UI, plus those of particular relevance to our study in terms of their function in relation to thermal stress, were further examined. To assess whether site, sampling time, or their interaction were significant in predicting relative lipid abundance, general linear models (glm) were used with lipid abundance as the response variable and site and time as the explanatory variables. Where there was no significant interaction between site and time, their effect on lipid abundance was plotted separately. Regressions and glm were carried out using SAS 9.4 (Cary, NC, USA).

To test whether occupation, here used as a proxy for mortality, was related to time, a logistic regression with a binomial errors was run for each site with occupied (1 or 0) as the response variable and time (days) as the explanatory variable.

## Additional Information

**How to cite this article**: Muir, A. P. *et al.* Lipid remodelling in the reef-building honeycomb worm, *Sabellaria alveolata*, reflects acclimation and local adaptation to temperature. *Sci. Rep.* **6**, 35669; doi: 10.1038/srep35669 (2016).

## Supplementary Material

Supplementary Information

Supplementary Dataset 1

## Figures and Tables

**Table 1:** Sampling locations spanning the latitudinal range of *S. alveolata* and their mean summer sea surface temperatures (°C; based on data from July–September 2013 taken from the NOAA World Ocean Altas^20^).

Site	Location	Temperature
Irish Sea	Auchenmalg, Scotland	13.73
English Channel	Mont St Michel, France	15.63
Bay of Biscay	Biarritz, France	20.71
Iberian Peninsula	Estoril, Portugal	18.65
North Africa	El Jadida, Morocco	23.02
