# Supplementary material for: Lipid remodelling in the reef-building honeycomb worm, Sabellaria alveolata, reflects acclimation and local adaptation to temperature
Source: Sci Rep. 2016 Oct 20;6:35669. doi: 10.1038/srep35669 (PMC5071839; doi:10.1038/srep35669)
Supplement: Supplementary Information [file srep35669-s1.pdf]

Lipid remodelling in the reef building honeycomb worm, *Sabellaria alveolata*, reflects acclimation and local adaptation to temperature

Muir, A. P., Nunes, F. L. D., Dubois, S. F. and Pernet, F.

Table S1: Results of a correlation matrix, which ranks lipids in explaining observed variation in the unsaturation index. These six lipids explain 98% of the observed variation.

Lipid	Rank	Partial R-squared	Model R-squared	F	P
20:5n-3	1	0.850	0.850	384.92	<0.0001
20:4n-6	2	0.056	0.905	39.33	<0.0001
Total DMA	3	0.028	0.933	27.69	<0.0001
18:4n-3	4	0.015	0.949	19.10	<0.0001
22:5n-3	5	0.020	0.968	39.50	<0.0001
22:6n-3	6	0.008	0.977	22.61	<0.0001
TOTAL		0.977			

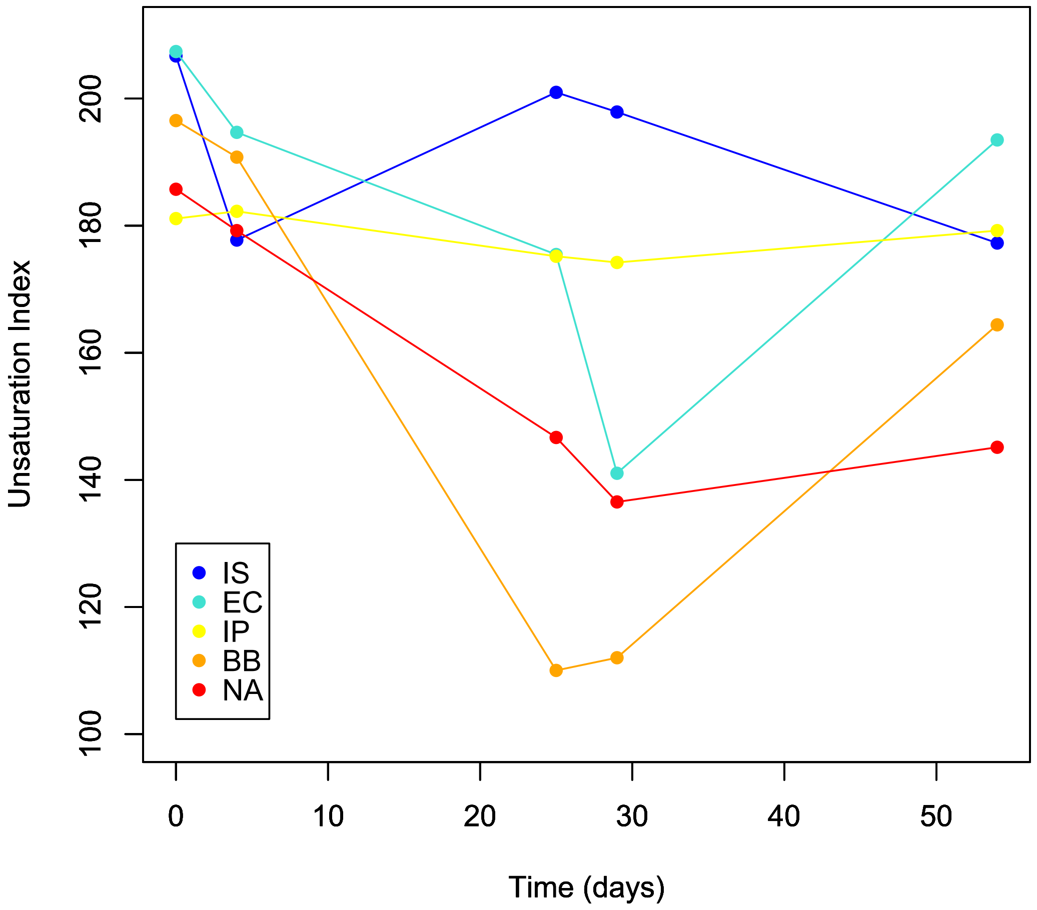

Figure S1: Plot showing the non-significant interaction effect of time and site on unsaturation index. The abbreviation IS is used for Irish Sea, EC for English Channel, IP for Iberian Peninsula, BB for Bay of Biscay and NA for North Africa.

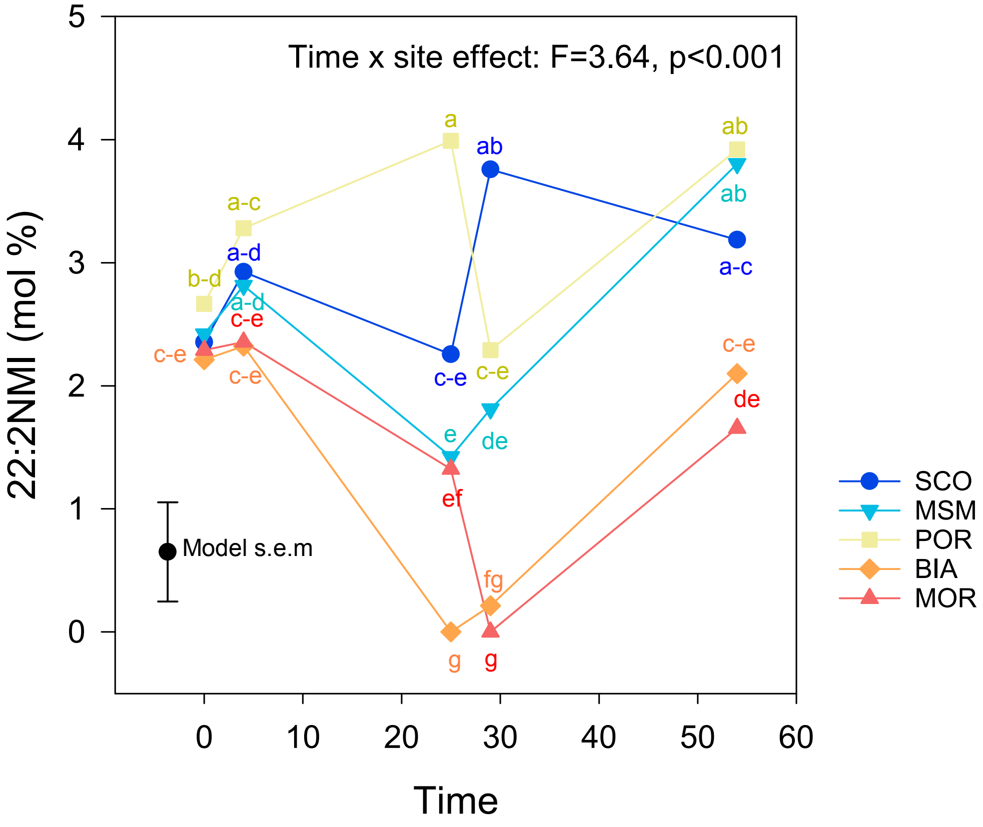

Figure S2: Plot showing the significant effect of the interaction between time and site on 22:2NMI. Letters are used to show pairwise significant differences such that if no letters are shared between two points then there is a significant difference between them ($p<0.05$). Standard error of the model is shown (Model s.e.m). The abbreviation IS is used for Irish Sea, EC for English Channel, IP for Iberian Peninsula, BB for Bay of Biscay and NA for North Africa.

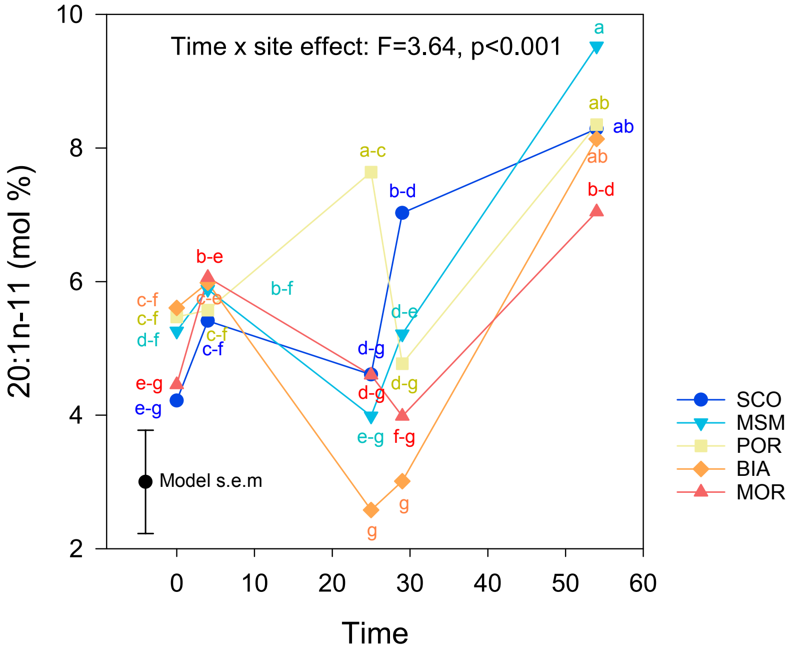

Figure S3: Plot showing the significant effect of the interaction between time and site on 20:1n-11. Letters are used to show pairwise significant differences such that if no letters are shared between two points then there is a significant difference between them (p<0.05). Standard error of the model is shown (Model s.e.m). The abbreviation IS is used for Irish Sea, EC for English Channel, IP for Iberian Peninsula, BB for Bay of Biscay and NA for North Africa.

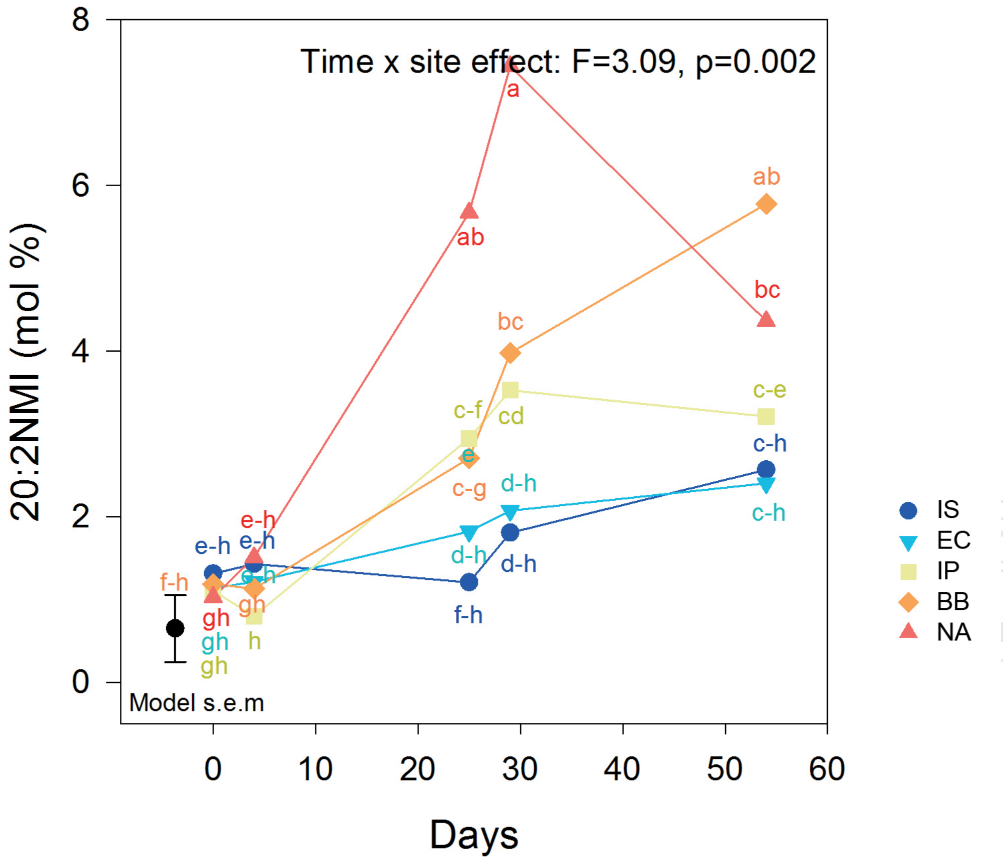

Figure S4: Plot showing the significant effect of the interaction between time and site on 20:2NMI. Letters are used to show pairwise significant differences such that if no letters are shared between two points then there is a significant difference between them ($p<0.05$). Standard error of the model is shown (Model s.e.m). The abbreviation IS is used for Irish Sea, EC for English Channel, IP for Iberian Peninsula, BB for Bay of Biscay and NA for North Africa.

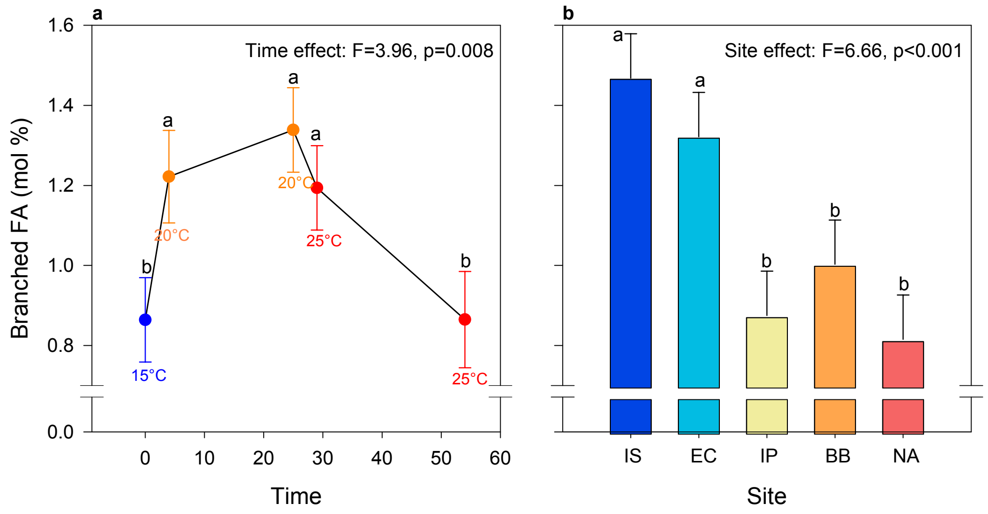

Figure S5: Plots showing the significant effect of time (days; a) and of site (b) on branched fatty acids. Letters are used to show pairwise significant differences between timepoints or sites such that if no letters are shared between two categories then there is a significant difference between them. The abbreviation IS is used for Irish Sea, EC for English Channel, IP for Iberian Peninsula, BB for Bay of Biscay and NA for North Africa. Sites are ordered from left to right on the axis from coldest to warmest.
